# Complete Genome Sequence of *Marinobacterium aestuarii* ST58-10^T^, a Benzene-Degrading Bacterium Isolated from Estuarine Sediment

**DOI:** 10.1128/MRA.00971-18

**Published:** 2018-09-20

**Authors:** Kyunghwa Baek, Seung Seob Bae, Jaejoon Jung, Dawoon Chung

**Affiliations:** a National Marine Biodiversity Institute of Korea, Seocheon-gun, Chungcheongnam-do, Republic of Korea; University of California, Riverside

## Abstract

Marinobacterium aestuarii ST58-10^T^ was identified as a benzene-degrading aerobic bacterium isolated from estuarine sediment in the Republic of Korea. The genome of strain ST58-10^T^ was found to be composed of a single circular chromosome (5,191,608 bp) with a G+C content of 58.78% and harboring 4,473 protein-coding genes. The assembled sequence data will help elucidate potential metabolic pathways and mechanisms responsible for the hydrocarbon-degrading ability of M. aestuarii ST58-10^T^.

## Abstract (precis)

Marinobacterium aestuarii ST58-10^T^ was identified as a benzene-degrading aerobic bacterium isolated from estuarine sediment in the Republic of Korea. The genome of strain ST58-10^T^ was found to be composed of a single circular chromosome (5,191,608 bp) with a G+C content of 58.78% and harboring 4,473 protein-coding genes.

## ANNOUNCEMENT

The genus Marinobacterium was proposed by Gonzalez et al. (1), with the description of Marinobacterium aestuarii as the type species. To date, 17 type strains from different species in this genus have been identified (http://www.bacterio.net/marinobacterium.html), and these strains have been isolated from various marine habitats. M. aestuarii ST58-10^T^ shows benzene-degrading activity of up to 70%, which was first observed within reported Marinobacterium species (2). To date, 10 genomes of Marinobacterium species have been published in the NCBI genome database; however, the genome of only M. aestuarii ST58-10^T^ has been sequenced completely. Considering the unique benzene-degrading properties of this strain and its dominant composition in hydrocarbon-rich environments, such as oil reservoirs (3, 4), we conducted genome sequencing to elucidate the physiological roles and metabolic potential of M. aestuarii ST58-10^T^.

The genomic DNA was purified from cells after overnight culture in marine broth 2216 at 25°C using an AccuPrep bacterial genomic DNA extraction kit (Bioneer, South Korea) according to the manufacturer’s instructions. The genome was sequenced from a 20-kbp library by using the single-molecule real-time (SMRT) sequencing method with a PacBio RS II system (Pacific Biosciences, USA). The 81,479 reads were assembled using Hierarchical Genome Assembly Process version 3 with default parameters. Genome annotation was performed using the NCBI Prokaryotic Genome Annotation Pipeline (5).

As a result of the final assembly, a single contig with an *N*_50_ value of 5,191,608 bp was generated, with coverage of 154× and 58.78% G+C content. In total, 4,473 protein-coding sequences were predicted, with 110 RNA genes, including 21 rRNAs (7 copies of 5S, 16S, and 23S), 85 tRNAs, 4 noncoding RNAs, and 51 pseudogenes. In the genome, we detected genes encoding mechanisms for xenobiotic biodegradation and metabolism-related enzymes, such as phenol hydroxylase and benzoate and/or toluene 1,2-dioxygenase. Many other aromatic hydrocarbon-metabolizing genes, such as protocatechuate 3,4-dioxygenase, anthranilate 1,2-dioxygenase large and small subunit, and haloacid dehalogenase, were also located on the chromosome. In addition, genes annotated as [2Fe-2S]-binding proteins, a component of the ring-hydroxylating dioxygenase alpha subunit responsible for the biodegradation of aromatic hydrocarbons, were identified (6).

In conclusion, the assembled genome sequence of *M. aestuarii* ST58-10^T^ presented here will contribute to the elucidation of regulatory pathways and metabolic networks involved in hydrocarbon degradation.

### Data availability.

Marinobacterium aestuarii ST58-10^T^ was deposited at the Korean Collection for Type Cultures (KCTC 52193^T^) and Biological Resource Center, National Institute of Technology and Evaluation (NBRC 112103T). The BioSample and BioProject accession numbers are SAMN04939310 and PRJNA320435, respectively. The complete genome sequence of Marinobacterium aestuarii ST58-10^T^ has been deposited at DDBJ/EMBL/GenBank under the accession number CP015839.
